# Setting a New Syllabus: Environmental Health Science in the Classroom

**DOI:** 10.1289/ehp.112-a814

**Published:** 2004-10

**Authors:** Valerie J. Brown

In the last few decades, the importance of the relationship between humans and the environment has become prominent in the social consciousness. Recognition of this importance has come from new understanding of environmental health issues, including evidence that many environment-related health problems, such as asthma and neurological damage from lead exposure, affect children disproportionately. A number of efforts are under way around the nation to educate children about the concepts and principles of environmental health with the goal of expanding science education, empowering children to avoid some adverse environmental exposures, and helping them to grow into informed citizens who can assess and affect important public health issues.

Development of curricula and teacher competency in environmental health for kindergarten through twelfth grade (K–12) began in the early 1990s. Since then, environmental health education has been implemented in many individual schools, but still has far to go to be adopted into the standard curricula followed by most school districts and states. For the most part, it is the initiative of individual teachers that brings environmental health into classrooms.

But while environmental health may not figure prominently on the radar screen of the educational establishment, education definitely figures on that of environmental health professionals. Institutions including the NIEHS, the NIH, the American Association for the Advancement of Science, and some museums have committed significant resources to creating environmental health curricula that may eventually become part of a standard education in the United States.

## Environmental Health: Health or Environment?

Although many, if not most, K–12 science curricula include environmental science components such as ecology and pollution remediation, few focus on environmental health. Environmental health is a multidisciplinary concept involving principles and methods from toxicology, epidemiology, endocrinology, public health, and other specialties. Whereas environmental science tends to address how human beings affect the rest of the biosphere, environmental health focuses on how the environment affects human health. Many environmental influences on human health are man-made—for example, pesticides, industrial chemicals, and air pollution—but environmental health also encompasses broad public health issues including tobacco use, infectious disease, indoor air quality and allergies, and sanitation.

Science and health tend to be separate tracks in typical curriculum frameworks. Marian Johnson-Thompson, director of education and biomedical research development at the NIEHS, says, “The likelihood of an environmental health course being taught is slim unless you’re at a specialized high school or a private school.” Moreover, says Lloyd Sherman, director of the Center for Excellence in Youth Education at Mount Sinai School of Medicine, if environmental health is taught, it’s usually an elective.

Although there are many professional and citizen groups in the United States devoted either to the environment or to education, not many are working on environmental health education per se. The National Environmental Education & Training Foundation, a nonprofit environmental literacy group in Washington, D.C., has issued a position statement advocating environmental health education, but it is aimed at raising awareness of environmental factors in health among medical clinicians, not teachers. A 2003 meeting report by the Washington, D.C.–based nonprofit National Council for Science and the Environment titled *Recommendations for Education for a Sustainable and Secure Future* advocated that “education for sustainability” be incorporated into six broad aspects of elementary and secondary education: teacher education; standards and assessment; community education; school partnerships and real-world knowledge; curriculum development and distribution; research; and funding. None of these six “essential learnings,” as the council calls them, specifically mentions environmental health, although environmental health could be incorporated into any or all of them. The State Education and Environment Roundtable (SEER), a consortium of 16 state departments of education, advocates using local natural and community surroundings as a context for learning in its “Environment as an Integrating Context” model of educational practices. But according to SEER director Gerald Lieberman, the consortium has not worked directly on environmental health education.

## Curriculum Conundrums

There are three realities of modern education that shape all public school classroom content: the National Science Education Standards (NSES), state curriculum standards, and the Elementary and Secondary Education Act, also known as the No Child Left Behind Act of 2001. Between them, these elements exert considerable influence over teachers’ options in teaching environmental health.

The NSES strongly influences science curricula and incorporates a variety of environmental principles. For example, one age-specific NSES benchmark is the requirement that K–2 students understand that “plants and animals need certain resources for energy and growth (e.g., food, water, light, air).” Students in grades 3–5 should know that “all organisms (including humans) cause changes in their environments, and these changes can be beneficial or detrimental.” A similar benchmark for grades 9–12 requires knowing “ways in which humans can alter the equilibrium of ecosystems, causing potentially irreversible effects,” for example through population growth, harvesting, pollution, and atmospheric changes.

The NSES thus sets broad goals that environmental health curricula can meet, but does not specifically mandate the teaching of environmental health topics. State curriculum standards are based—sometimes loosely, sometimes rigorously—on the NSES benchmarks. Like those benchmarks, state curriculum standards tend to require broad general skills rather than detailed subject skills.

No Child Left Behind is a different matter. It requires that students pass competency tests in basic skills, starting with reading and mathematics. Laura Hemminger, codirector of tthe Resource Center of the Environmental and Occupational Health Sciences Institute at the University of Medicine and Dentistry of New Jersey, says, “With No Child Left Behind, there’s this feeling that, for whatever is used in the classroom, there has to be a direct link to improving test scores.”

Dina Markowitz, director of the Center for Science Education and Outreach at the University of Rochester, agrees. “Mandated testing drives the lesson plans,” she says. Markowitz administers an NIEHS Environmental Health Sciences as an Integrative Context for Learning (EHSIC) grant as well as grants from several other sources to develop multidisciplinary material for K–12 teachers [for more information on EHSIC grants, see “Mission: Educational,” p. A806 this issue]. She believes the burden of required standardized testing is a barrier to developing usable environmental health curricula. She has had what she describes as “heated” discussions with federal education officials about it. She has told them that she is very grateful for the federal funding that allows her program to create unique curriculum units, but that testing requirements discourage teachers from using them.

Part of teachers’ nervousness about straying from the test subjects stems from No Child Left Behind’s provision that schools whose students do not show adequate progress are placed on a special list with annually progressive consequences for continued poor showings. No Child Left Behind can also introduce a skew by grade level into school curricula. Nancy Moreno, an associate professor of family and community medicine at Baylor College of Medicine Center for Community Outreach in Houston, says that No Child Left Behind requires only certain subject tests in certain years, and teachers increasingly tend to use class time to prepare students for specific tests.

“The emphasis on standardized testing is leading to concentrations of subjects in grade levels where there’s a mandated assessment,” Moreno says. “When the emphasis is on reading, language arts, and mathematics—which of course are important—the time allocated to science can end up being reduced.” On the other hand, Moreno says, in places where absolutely no science was being taught at all, children are enriched by the presence of required science testing in certain grades.

A further issue, primarily in elementary grades, is that many teachers are reluctant to teach science because they have little or no science background themselves. At the high school level, most science teachers do have at least some science background and are more autonomous in their teaching decisions, Moreno adds.

For those K–12 teachers who do have the interest and time to develop their own environmental health curricula, there are many materials available from government programs and other sources, especially on the Internet. For example, the National Library of Medicine Tox Town website (**http://toxtown.nlm.nih.gov/**) introduces visitors to basic toxicology concepts, shows where toxic chemicals might be located in a town or city, provides information about specific chemicals, and links to a database where visitors can learn whether their own communities have such chemicals in them. Tox Town is aimed at high school and college students, according to Cynthia Love, a technical specialist with the National Library of Medicine in Bethesda, Maryland.

The U.S. Environmental Protection Agency sponsors the SunWise Program (**http://www.epa.gov/sunwise/**), which suggests ways for teachers to convey health risks associated with sun exposure to children in grades K–8. SunWise offers a “toolkit” of lesson ideas, a website including a UV Index with information about the dangers of various degrees of UV exposure, a video, and other materials.

Another excellent resource is Science NetLinks, a website created by the American Association for the Advancement of Science and the MarcoPolo Education Foundation, a project of the telecommunications company MCI conducted as part of the still larger Internet Content for the Classroom project by a consortium of international education organizations and the MCI Foundation. The MarcoPolo site offers teachers free grade-specific web-based lessons and newsy reports on such topics as the Asian “brown cloud” of particulate pollution over the Indian Ocean. The lessons have been structured for easy adaptability to individual state standards. The site also links to other online resources screened for reliable and appropriate content.

But few teachers have the time to sort through the plethora of environmental health information to find not only educational materials but also those at an appropriate level of difficulty and content. Even if environmental health education were mandated at the school district or state level, most teachers would be hard-pressed to take on the extra work. Markowitz says Rochester public school teachers are already overwhelmed by the standard curriculum in a district that is the third largest in the state and one of the poorest. “At Rochester we can’t possibly mandate another thing to our teachers” without making sure the new material addresses existing curriculum requirements, she says.

“The trick is making [an environmental health] curriculum fit the requirements and making it easy on the teachers so that it’s something they can implement in the classroom very quickly,” says Sarah Weppner, environmental health education and assessment program director at the Idaho Department of Health and Welfare. Weppner heads a small educational outreach program funded by the Agency for Toxic Substances and Disease Registry (ATSDR) aimed at increasing community awareness of health risks associated with Superfund and other hazardous waste sites in Idaho. Her program has produced lesson plans and sponsors an annual essay and poster competition for middle school students.

Fortunately for those teachers who do have relevant methods and materials and are comfortable using them, state standards are often broad enough that environmental health can be used to fulfill some science and health curriculum requirements. In New York, says Marissa Maggio, who teaches environmental health in that state, it’s relatively easy to meet the state standards, at least at the secondary level. “For anything you do, you can find a standard that fits what you’re teaching, and you can teach beyond what standards require,” Maggio says.

## K–12 Initiatives

Because of the constraints on teaching science, an effective strategy for designing a K–12 environmental health curriculum is to make it kill several birds with one stone—that is, to structure it so that it challenges students not only in environmental science and health, but also in reading, mathematics, social studies, and other standard subjects. Moreno has found that when she and her team present this integrated multidisciplinary approach to teachers at professional development workshops, “they’re kind of surprised. Once they start thinking about it, they find it fits in with what’s happening in the classroom.”

The NIEHS has been a pioneer in supporting K–12 environmental health education, including development of interdisciplinary curricula through its EHSIC grants. Johnson-Thompson says when she joined the NIEHS in 1992, director Kenneth Olden asked her to immediately address how the institute could develop a K–12 science educational activity. Initially, she says, the program focused on getting more students interested in science with the goal of fostering the next generation of scientists. The program later took on the additional focus of ensuring that all children become scientifically literate citizens.

In 1993 the NIEHS issued its first request for applications to develop environmental health curricula. Moreno and Hemminger were among the early recipients of NIEHS funding. Hemminger’s program developed a curriculum for grades K–9 called ToxRAP to teach environmental health using risk assessment concepts, while Moreno’s group developed a package of environmental health educational materials for grades 2–4 called My Health My World. This package integrates environmental health topics such as infectious disease and chemical pollution with language arts, math, and other standard curriculum subjects, meeting science education standards along the way. Today the NIEHS continues to fund a variety of environmental health education projects [for more information on these programs, see “Mission: Educational,” p. A806 this issue].

Science-based activities for elementary school children are notoriously scarce; where possible, their teachers often turn to museums for learning opportunities. Moreno’s program at Baylor teamed up with the Children’s Museum of Houston to create a traveling exhibit called My Home Planet Earth, which is based on the My Health My World curriculum. Funded by the NIEHS and the NIH Science Education Partnership Award program of the National Center for Research Resources, the exhibit is bilingual in Spanish and English. It will be at the Children’s Museum of Seattle until January 2005.

Chris Cooper, director of external affairs at the Seattle museum, says the exhibit “is a terrific match for our audience.” The exhibit features the characters Riff and Rosie, who are squirrels, and Mr. Castor Slaptail, a professorial beaver. Riff and Rosie introduce children to issues such as allergy triggers and indoor air quality, eutrophication and toxic waste in water supplies, and microbial activity in leftover foods in the refrigerator. Along the way the children learn scientific concepts: a jar full of 999,999 yellow cupcake sprinkles and a single black one illustrates parts per million; an “achoo” pinball game shows how particulate matter enters lungs.

The most popular activity is a Rube Goldberg–like contraption full of blue balls representing clean water. To learn how pollutants behave, children can put slightly smaller brown balls in the machine, which other children try to remove before they reach the main water supply. “Kids just love it,” Cooper says. “A good exhibit should hold kids’ attention for twenty minutes. We have kids playing [at the ball machine] for an hour and a half.” Cooper adds that during the summer the exhibit contributed to a 150% increase in group visits to the museum over the year before, and he expects school groups during the academic year to pick up considerably as well.

## The Secondary Level

At the secondary level there are also very few established environmental health programs, but one shining example stands out at the High School for Environmental Studies (HSES) in New York City, a specialty secondary school focusing on the environment. The program has existed since 1994 as a collaboration with the Mount Sinai School of Medicine environmental health outreach program, which is directed by Sherman. It is an outgrowth of Mount Sinai’s Superfund grant to study organochlorine contaminants in the Hudson River. The high school’s collaboration with Superfund investigators has resulted in the creation of the environmental health curriculum, which has been adopted by the high school as an elective.

Maggio teaches the environmental health course, and helps implement a zebrafish research project and a host of weekly activities that bring students and Superfund investigators together in this year-round program to increase environmental health literacy among inner-city students. Because classes are small and the health aspect is already embedded in a general environmental science context, Maggio has the luxury of focusing on advanced knowledge, such as epidemiology and toxicology, to a far greater extent than most high school teachers can manage.

In its entrance requirements, HSES relies as much on student interest as it does on specific knowledge or skill levels, according to Sherman. HSES students don’t take tests. Instead, Maggio requires them to do research projects in which they search the scientific literature, write reports including proper citation of their sources, and present results to the class. “I have seventeen-year-old students reading peer-reviewed journals,” Maggio says proudly.

The program is structured so that the students learn to do investigative work themselves. Jennifer Vasquez, a 2003 graduate of HSES and now a sophomore at Connecticut College, focused on endocrine disruption in the health class. She says Sherman and Maggio inspired and challenged her. “They forced you to learn [by] yourself. It’s actually better that way, and it sticks with you,” she says. Vasquez is considering an environmental studies major.

Vasquez, senior Jamie Ahn, and several other students have spent the last two years developing a zebrafish breeding system. This summer they conducted their first experiment with the fish by exposing them to DDT. They conducted a literature review, kept a lab log, and presented their results to a group of Mount Sinai doctors, reporting that DDT exposure affected both zebrafish reproduction and survival, and that it may affect the fishes’ swim bladders as well.

The HSES environmental health program has been successful enough that Maggio is now getting calls from teachers at other New York City schools who are interested in teaching the subject, and a second environmental high school recently opened in the city. At the start of the 2004 HSES school year, 26 students had enrolled in the environmental health course, up from 18 last year.

Environmental justice is another facet of environmental health that is taught at HSES; it is part of the school’s environmental ethics class, which is required for juniors. The HSES student body is highly multicultural, with many disadvantaged and minority students for whom health disparities are a personal matter. The students become aware of environmental justice issues directly relevant to themselves, their families, and their friends, and Maggio says the subject is one of their “favorite issues to talk about in ethics class.” For example, in studying the Super-fund problems in the Hudson River, they learn that the river’s contamination affects poor and minority residents disproportionately because those populations eat the fish they catch. During literature searches, Maggio adds, the students often discover articles relating to their own neighborhoods.

Because of this immediacy, Sherman says, “kids get up at six in the morning and don’t leave until six at night—because everything they study has meaning. When education connects with that, there’s no stopping it. The appetite is sensational.”

Ahn, who hopes to pursue a career in environmental toxicology or environmental policy, appreciates the way environmental health brings its related issues and concepts very close to home. “I have applied my knowledge to my and my family’s life,” she says. “I have learned about the dangers [from polychlorinated biphenyls] of eating fish from the Hudson River, and the effects of industrial [toxicants] on human health.”

Vasquez was surprised to learn of the health threat to Hudson anglers, who tend to be minorities, poor, or immigrants. But she says the ethics class and her study of endocrine disruption have empowered her to believe she can make a difference in the world and that “it’s cool to know you can help.”

Across the state, in Rochester—where childhood lead poisoning is a huge problem and many neighborhoods are poor—Markowitz’s program incorporates social studies as focused through the environmental justice lens to get students asking questions about health disparities. One alternative high school she works with is built on a former auto repair shop across the street from a landfill. A science teacher there introduced the issue of indoor air quality in the school itself as an environmental health education topic. Soon the students—whom the teacher had told Markowitz were “reluctant readers”—were enthusiastically using Internet search engines to gather data about indoor air quality, studying websites, and presenting information to each other in class. According to Markowitz, the teacher was able to take the time for this project in part because the school is an alternative one whose students are exempt from the testing mandated by No Child Left Behind. Another Rochester teacher has used environmental health as subject matter in an English as a Second Language class.

## Graduating to the Next Level

Hard numbers about the extent to which environmental health is being taught in grades K–12 nationally are not available. Hemminger estimates that about 4,500 teachers in 23 states are using the ToxRAP materials. Moreno says she and her colleagues have trained more than 5,000 teachers, representing almost every U.S. state, in the My Health My World curriculum. Weppner believes about 500 students in two schools participated in the 2004 Idaho State/ATSDR essay and poster contest. Sherman estimates that between 120 and 200 students in all have passed through the HSES environmental health program.

While the NIEHS has perhaps the most broad-based approach, there are also many more narrowly focused resources available to educators. Taken together, these initiatives are making it easier for teachers who want to teach environmental health to find curriculum ideas, lesson plans, professional development, and ways to use environmental health to teach mandated subject areas.

There are no nationwide curriculum requirements in place for environmental health, but there are pockets where it is being taught, usually in environmental science classes; a very few specialized secondary schools place more emphasis on it. But environmental health education experts believe that if environmental health topics can be integrated with standard curriculum requirements, especially through multidisciplinary projects, they are much more likely to be used by teachers already laden with state curriculum standards and the strictures of the No Child Left Behind Act. And while formal incorporation into standard school curricula is still some distance off, Sherman says, there is no way that environmental health can not be part of the continuing educational narrative, because people everywhere are making the connection between human diseases and environmental factors. “Environmental health education,” he says, “is a genie you cannot put back in the bottle.”

## Figures and Tables

**Figure:**
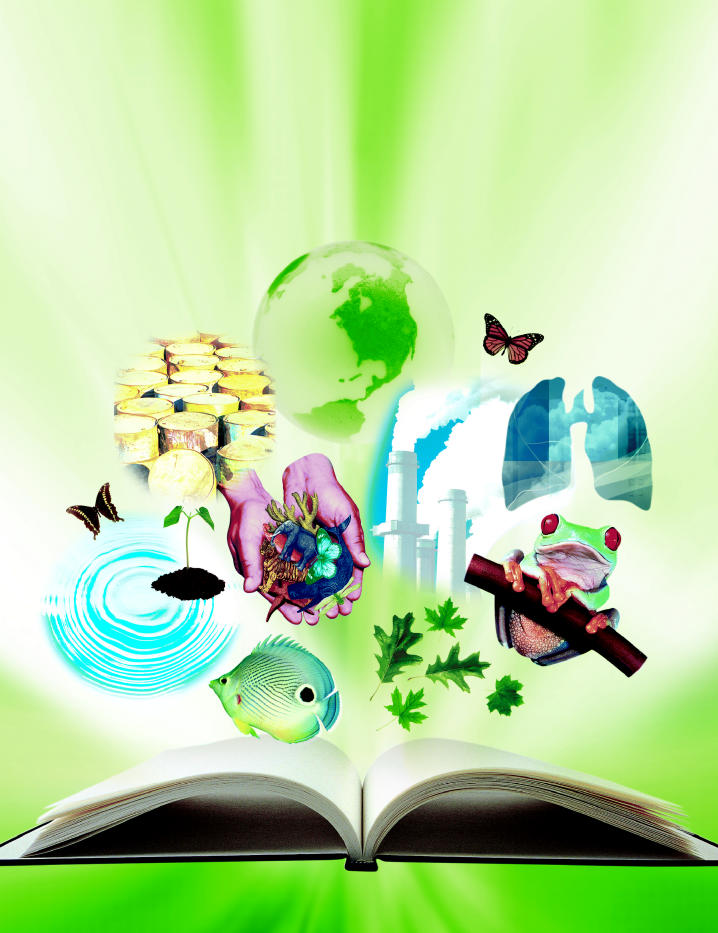


**Figure:**
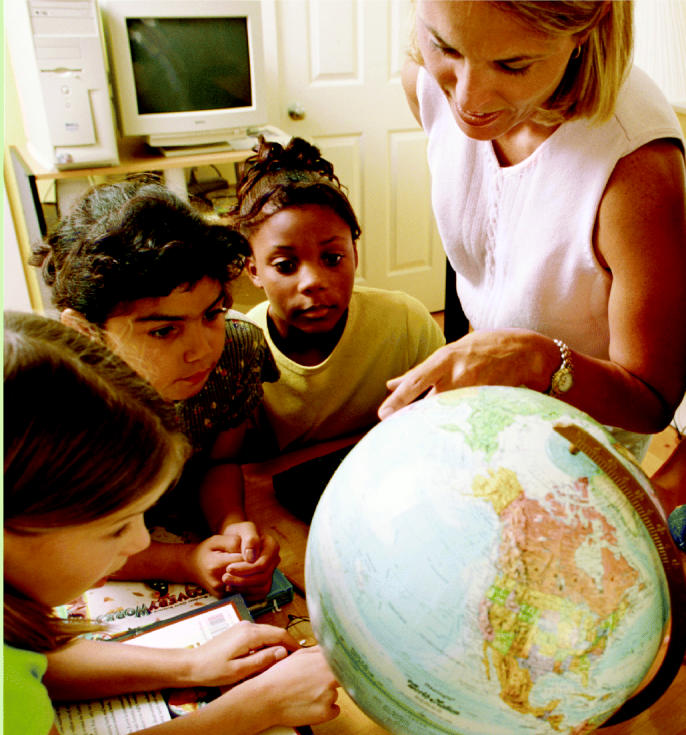
**A world of opportunities.** Curricula that use environmental health as a teaching model can give children a global perspective on science.

**Figure:**
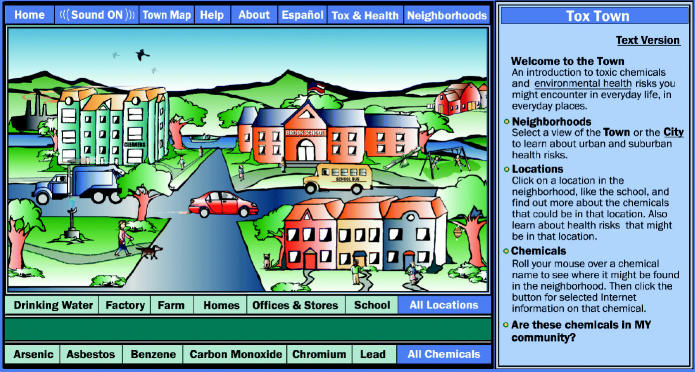
**Bringing the message home.** The National Library of Medicine’s Tox Town website encourages students to investigate chemical exposures in their own communities.

**Figure:**
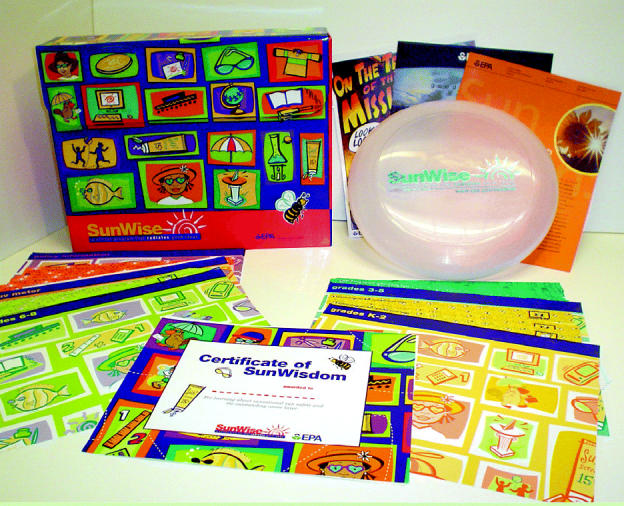
**Bright idea.** The Environmental Protection Agency’s SunWise program educates students on the dangers of sun exposure and gives them concrete steps they can take to protect their own health.

**Figure:**
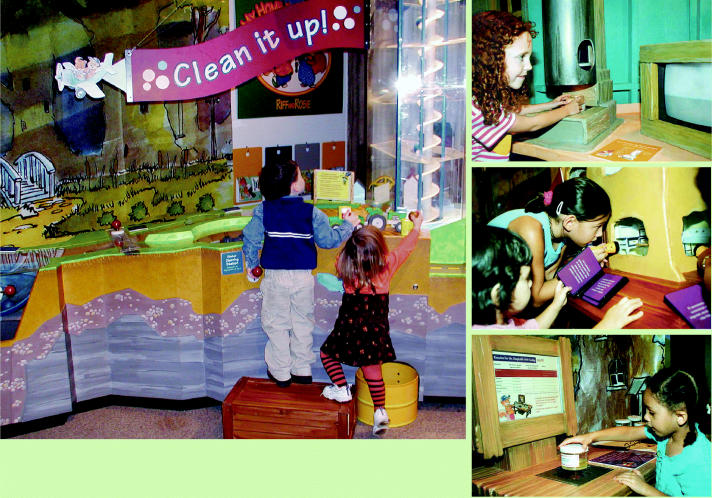
**Hands-on learning.** The traveling museum exhibit My Home Planet Earth teaches children about environmental health topics such as (clockwise from left) remediation (“Clean It Up!”), research (“Video Microscope”), indoor air exposures (“Allergen House”), and pollution (”Mucky Water”).

**Figure:**
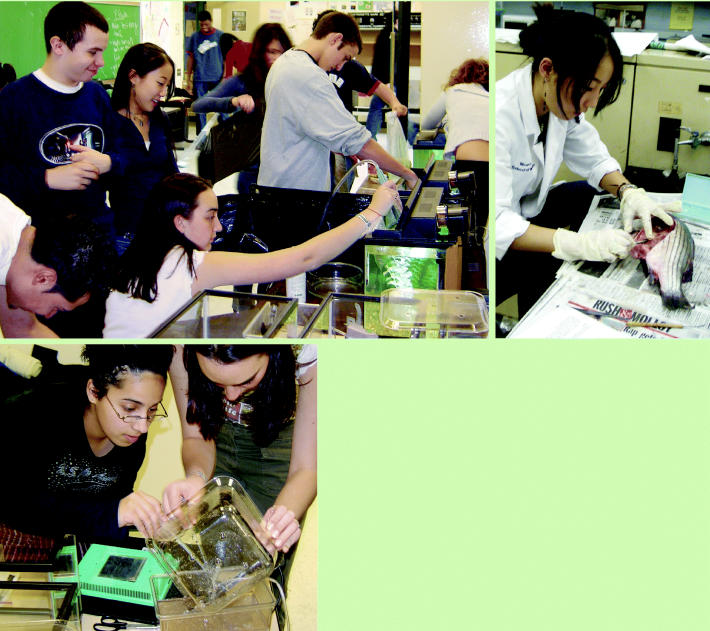
**Scientists in the making.** Students at the High School for Environmental Studies learn by conducting actual environmental health experiments. Clockwise from left: Gabrielle Torres and Gabrielle Niccolls harvest brine shrimp, which were hatched to feed baby zebrafish; students set up the zebrafish lab at school; Jamie Ahn dissects a striped bass, a fish native to the Hudson River.

